# Soft Actuated Hybrid Hydrogel with Bioinspired Complexity to Control Mechanical Flexure Behavior for Tissue Engineering

**DOI:** 10.3390/nano10071302

**Published:** 2020-07-03

**Authors:** Ramón Rial, Zhen Liu, Juan M. Ruso

**Affiliations:** 1 Soft Matter and Molecular Biophysics Group, Department of Applied Physics, University of Santiago de Compostela, 15782 Santiago de Compostela, Spain; ramon.rial@usc.es; 2 Department of Physics and Engineering, Frostburg State University, Frostburg, MD 21532, USA; zliu@frostburg.edu

**Keywords:** hydrogels, scaffolds, bionanoparticles, tissue engineering, mechanical properties

## Abstract

Hydrogels exhibit excellent properties that enable them as nanostructured scaffolds for soft tissue engineering. However, single-component hydrogels have significant limitations due to the low versatility of the single component. To achieve this goal, we have designed and characterized different multi-component hydrogels composed of gelatin, alginate, hydroxyapatite, and a protein (BSA and fibrinogen). First, we describe the surface morphology of the samples and the main characteristics of the physiological interplay by using fourier transform infrared (FT-IR), and confocal Raman microscopy. Then, their degradation and swelling were studied and mechanical properties were determined by rheology measurements. Experimental data were carefully collected and quantitatively analyzed by developing specific approaches and different theoretical models to determining the most important parameters. Finally, we determine how the nanoscale of the system influences its macroscopic properties and characterize the extent to which degree each component maintains its own functionality, demonstrating that with the optimal components, in the right proportion, multifunctional hydrogels can be developed.

## 1. Introduction

Finding new materials with the suitable properties to be used as extracellular matrix (ECM) substitutes has always been one of the major goals of the tissue engineering. In recent years, many works have focused on the study of polymeric hydrogels, since their properties of biocompatibility, biomimicry, receptivity, the possibility to adjust their mechanical properties, and their intrinsic ability to contain great amounts of water make them excellent substitutes of the ECM for biomedical applications [1,2,3,4,5]. Consequently, naturally derived polymeric hydrogels present themselves as flexible and adaptable scaffolds to mimic the natural features of native ECM such as the stimulation of tissue formation and maintain and conserve cellular functions [6]. Another important point is the interaction between these scaffolds and proteins of the hosting tissue [7,8]. This process is crucial defining whether the treatment is going to be effective. In this regard, the mechanisms and the attributes that affect to the protein adsorption and interactions on the compounds are highly difficult to characterize [9,10,11]. Nevertheless, in order to design new materials or devices to be used in the regeneration of calcified tissues, it is of vital importance to understand the processes, that is the reason why this topic has been one of the major concerns in the field of regenerative medicine over the recent years [12]. In this regard, polymeric scaffolds have been recently used [13,14] to bioengineer 3D cell culture studies, as well as developing tissue formation ex vivo and cellular organization. However, the possibilities are not only limited to this field, excellent reviews have shown the great versatility of these compounds. Sanchez et al. [15], in addition to analyzing the most popular synthesis routes, described some of the most surprising examples of applications, both commercial and prototype. The combination of the properties of organic (stabilization, softness) and inorganic compounds (strength, conductivity) is the key of hybrid materials for optics, bioimaging, plamonics, electronics, and storage [16]. The perfect synergy of the complementary properties of both components is also highlighted by Saveleva et al. [17], with an emphasis on the organization of the compounds (organic-in-inorganic and inorganic-in-organic). In order to produce hydrogels, the most typical and frequent materials used are biopolymers. Their properties and possibilities are so diverse that hydrogels have been used in recent times in a wide variety of possibilities, such as cell delivery, encapsulation systems, injectable materials, scaffolds, and even as bioink for biofabrication [18]. However, pure or one-compound hydrogels are subject to significant limitations and their applications are restricted [19,20]. The combination of different biopolymers provides a useful and encouraging mixture of cell health promotion given by the proteins and the good mechanical properties provided by the polysaccharides.

Albumin is the most abundant extracellular protein; maintaining colloidal osmotic pressure in plasma, carrying insoluble components and is the major calcium-binding protein present in the blood exhibiting up to 19 calcium binding sites on its imidazole groups. Also, it is known to be the first to surround foreign bodies when they are in contact with blood passivating their surfaces, blunting both proinflammatory and thrombogenic responses. Previous studies also remark that albumin is the main protein involved in the bioactive glass adsorption and a potent facilitator of mesenchymal stem cells colonization on mineralized human bone allografts [21]. On this line, fibrinogen has become an attractive material to be used in tissue engineering because of its moderate cost and its versatility to produce a wide range of scaffolds. This protein is naturally present in blood plasma as a zymogen, but when an injury occurs, it is stimulated by multiple reactions to evolve into fibrin fibers, essential in the wound coagulation process [22]. On the other hand, fibrin is naturally generated by the human body and its function is to act as a provisional scaffold for healing and regeneration [23]. Jockenhoevel et al. [24] have demonstrated that fibrin gels present a great combination of attributes of an optimal scaffold to allow the cell growth and the development of several tissue constructs. This protein, nonetheless, present an important lack in mechanical properties, so it is very common to combine it with polysaccharides [25,26,27].

Alginate is a polyanion composed by two monomeric units: β-D-mannuronate (Munits) and α-L-guluronate (Gunits) and linked by β(1–4) bonds [1,28,29] and its use in tissue regeneration has become also very popular, because of its natural ability to form different gels and its biocompatibility that is used for interfacing with cells [30]. Nevertheless, its hydrophilicity leads to poor protein adsorption, and its high molecular weight to low biodegradability [31]. Those drawbacks have restricted its functionalities and therefore its applications to a certain extent. However, combination with proteins completely solves this problem [25,26,32]. Gelatin is the product resulting from the denaturalization of the collagen protein, therefore, they present some differences in their biological and physical properties, such as cellular binding behavior and mechanisms [33] or gel point temperature [34]. The combination of alginate and gelatin provides a very propitious material to use as a substitute of the extracellular matrix (ECM) because of its chemical affinity. Various studies have also shown the advantages and application of hydrogels combinations [35,36,37,38]. Besides, fibrinogen and gelatin hydrogels have the capability of stimulate in vitro osteogenic differentiation of adipose stem cells or reinforce chondrogenesis of bone-derived mesenchymal stem cells [39]. Analogously, similar hydrogels have included gelatin for the synthesis of biomimetic scaffolds used in tissue engineering [40] or for producing tubular constructs mimicking blood vessels [41].

Finally, hydroxyapatite (HAp), the main component of bones and teeth, is a highly biocompatible and very economical product because of its high availability. Recently, beyond its applications in bone regeneration, the rise of biotechnology has increased interest in these biomaterials for new applications. These facts have made hydroxyapatite one of the most attractive and important bioceramics [42].

Based on these ideas, we propose the design and develop of a multi-component hybrid hydrogel composed of gelatin, alginate, hydroxyapatite, and protein (fibrinogen or bovine serum albumin). The components were chosen in function of the above explained features and based on our previous experience working with materials in the regenerative medicine area. Notably, this study implies a significant novelty in the field, as no similar gels with this complexity have been designed or characterized before. We hypothesized that cell interaction can be established by functionalizing the coatings with proteins [43], while particles in hydrogel coatings can serve for establishing focal adhesion [44,45].

The nano- and micro- characterization of the hydrogels has been performed by means of FT-IR and confocal Raman. Then, degradation studies and swelling properties were also performed in order to determine the system with the best properties. Finally, rheological measurements (flow curves, oscillatory sweeps, creep, and thixotropy test) were extensively used to obtain information about the mechanical properties, the internal structure of the samples, the resistance to deformation, and its response or the breakdown and eventual buildup of the microstructures of all samples under study.

Overall, this work contributed a new insight into the interactions between multicomponent hydrogels based on quantitative rheological methods reflecting the different rheological properties and the results obtained should be of great utility in the extensive application of hydrogels for using in tissue engineering.

## 2. Materials and Methods

### 2.1. Reagents

Hexadecyl-trimethyl ammonium bromide (CTAB, MW = 364.48 g mol^−1^, 99% Sigma-Aldrich, Madrid, Spain), poly (propylene glycol) (PPG, Sigma-Aldrich, MW = 425 g mol^−1^, δ = 1.004 g cm^−3^ at 25 °C), sodium phosphate (Na_3_PO_4_, MW = 148 g mol^−1^, 96% Sigma-Aldrich), calcium chloride (CaCl_2_, MW = 91 g mol^−1^, 99% Sigma-Aldrich), sodium nitrite (NaNO_2_, MW = 69 g mol^−1^, 97%), commercial alginic acid (Alg, from brown algae, ref. A7003, average MW = 120,000~190,000 g mol^−1^) from brown algae, and gelatin from bovine skin (gel strength~225 g Bloom, Type B, average MW = 40,000~50,000 g mol^−1^) were purchased from Sigma-Aldrich and were used for the synthesis of the samples directly without purification. For solution preparation, only triple -distilled water was used.

### 2.2. Preparation of HAp Nanorods

Hydroxyapatite nanorods were synthesized following an adaptation of the procedure proposed by Liu et al. [46]. The methodology can be described in three different steps:

Step 1. *Micellar solution preparation.* First, proper amount of commercial CTAB was weighed and dissolved in 35 mL of distilled water until reaching a 3.13 mM solution. Then, 2 mL of polypropylene glycol (PPG) were added to the mixture, put in an autoclavable bottle and stirred for 10 min; the addition of CTAB and PPG creates a micellar solution with a high degree of ionization. Above the critical micellar concentration, CTAB turns spherical micelles into rod-like shapes. As the nucleation of HAp occurs at the interface of the micelelles, the mixture serves as a template which give to the nanoparticles their final size and shape.
Step 2. *Chemical Reaction.* Next, an aqueous solution of 2 M NaNO_2_ and 0.22 g of CaCl_2_ were added and stirred until complete dissolution, obtaining the calcium precursor. After that, 20 mL of Na_3_PO_4_ 140 mM were added drop by drop to the shaking solution. At this point, phosphate groups interact with the calcium ions at the outer part of the micelles, starting the chemical reaction and nucleation. After all the reagents were combined, the stirring was maintained for 60 min.
Step 3. *Filtration, thermal processing and purification.* After the reaction, the solution was autoclaved at 120 °C for 24 h. Following, the mixture was filtered through a moistened, fat-free, double filter paper, and washed with 200 mL of distilled water. After that, in order to eliminate the excessive moisture of the samples, they were dried in an oven at 50 °C for 24 h. The final step consisted in a thermal treatment to eliminate completely the possible impurities that the sample could present, igniting it in the muffle furnace at 400 °C for 3 h.

### 2.3. Preparation of the Crosslinked Films

Step 1. *Preparation of the protein solutions.* To synthesize the hydrogels, each one of the protein solutions was prepared separately. First, a known amount of the alginic acid sodium salt powder was dissolved in phosphate buffered saline (PBS) to obtain a 5% (*w*/*v*) solution. The mixture was then kept under gentle agitation at 250 rpm during 3 h at 37 °C. Next, and similarly, a 2% (*w*/*v*) gelatin solution was prepared by dissolving the gelatin powder in PBS. The solution was magnetically stirred at 300 rpm and 58 °C for 1 h. Meanwhile, both fibrinogen and bovine serum albumin 0.5% (*w*/*v*) solutions were prepared by dissolving the proper amount of each one of the materials in the buffer and agitated until complete dissolution.
Step 2. *Obtention of the protein mixtures.* When all the solutions were ready, alginate was combined with fibrinogen or bovine serum albumin, respectively, at a 1:1 ratio, to obtain the initial Alg/Fib and Alg/BSA mixtures. Then, the gelatin was added to both mixtures at a volume ratio of 2:1:1 (gelatin: alginate: Fib/BSA) and stirred gently until getting the homogeneous viscous solutions Gel/Alg/Fib and Gel/Alg/BSA.
Step 3. *Formation and drying of the films*. Once the final protein mixtures were obtained, aliquots of 4.5 mL were added to 35 mm diameter Petri dishes and left to dry for 24 h at 45 °C. When the protein mixtures are appropriately dried, as a consequence of the great loss of liquid they suffer in the oven, they become fragile films that lay in the bottom of the Petri dishes (Gel/Alg/Fib and Gel/Alg/BSA films).
Step 4. *Preparation of the crosslinking solutions.* Meanwhile, two different fresh crosslinking solutions (C1 and C2) were prepared in Falcon tubes. The first crosslinking solution C1 was composed of 3.48 mL of a 110 mM aqueous solution of CaCl_2_, 7.08 mL of 11 mM HAp solution, and 7.44 mL of water. Crosslinking solution C2 was prepared similarly but without the presence of HAp: 3.48 mL of 110 mM of CaCl_2_ and 14.52 mL of water. The addition of CaCl_2_ assured the alginic acid polymerization and the crosslinking by calcium diffusion through the gels.
Step 5. *Crosslinking process and obtention of the final hydrogels.* At this point, Falcon tubes containing the crosslinking solutions were sonicated for 10 min and vortexed. Finally, 1.5 mL of the crosslinking solutions C1 and C2 were added into empty Petri dishes. Dried protein films were subsequently placed on top of the solutions using tweezers, carefully enough to avoid wrinkles and bubbles in the membranes. Next, films were left on an orbital shaker at 150 rpm at room temperature (RT) for 3 h, to ensure a complete crosslinking. Following this procedure, each of the films crosslinked with the C1 solution contained a final amount of HAp of 6.525 mg.

Hydrogels resulting from the crosslinking with the C1 solution were named Gel/Alg/Fib/HAp and Gel/Alg/BSA/HAp and the ones crosslinked with C2, Gel/Alg/Fib and Gel/Alg/BSA. Furthermore, in order to explore the influence of fibrinogen and bovine serum albumin on the properties of the hydrogels, a blank sample Gel/Alg/HAp was also prepared following the explained methodology and crosslinked with the solution C1.

### 2.4. Characterization of the Polymeric Hydrogels

*FT-IR spectroscopy*. For the Fourier transform infrared spectroscopy (FT-IR), gels were dried and placed on a microsample cup. Data acquisition was performed using a FT-IR spectrometer (Varian FT-IR 670, Agilent, Santa Clara, CA, USA) coupled to a mapping microscope (Varian620-IR, Agilent, Santa Clara, CA, USA). Samples were studied in the interval 400 to 4000 cm^−1^ with a spectral resolution of 4 cm^−1^ and 64 scans∙min^−1^.

*Raman microscopy*. Spectroscopy data of the gels were obtained with a WITec Confocal Raman Microscopy model Alpha 300R+y (Ulm, Germany). Dried samples were partially wet with triple distilled water to homogenize them and to avoid fluorescence. In a typical experiment, a frequency doubled laser at 532 nm of excitation wavelength with an output power of 38 mW was used. Spectra were recorded using a 50× Zeiss, EC Epiplan-Neofluar Dic objective (Oberkochen, Germany) with a numeric aperture of 0.8. Signals were detected in the range of 1024 × 127 pixels. The number of accumulations was 30 and the integration time per pixel was 0.2 s. Data acquisition was driven by the WITec Control software (Ulm, Germany).

*Swelling behavior.* The swelling and degradation characteristics of the polymeric hydrogels were performed in phosphate buffered saline (PBS) with pH 7.4 at 37 °C. For the swelling studies, samples were dried and weighed (*W_d_*), then they were immersed in PBS and taken from the solution at selected time intervals of 0.5, 1, 3, 6, 8, 12, 24, 48, and 72 h, wiped with blotting paper, weighed (*W_s_*) and placed again in PBS. For the degradation, hydrogels were synthesized, weighed (*W_d_*) and also placed in PBS. In contact with the buffer, hydrogels suffer a modification in their structure, starting the release of material to the medium. At the specific timepoints, samples were removed from release medium and weighed after absorbing water on the surface with filter paper (*W_r_*). The swelling ratio (SR) and degradation ratio (DR), in percentage, were calculated gravimetrically by the following equations:

(1) $$\mathrm{Swelling}\;\mathrm{Ratio}\;\left(\mathrm{SR}\right)\;\left(\%\right)=\frac{W_{s}-W_{d}}{W_{d}}\times 100$$

(2) $$\mathrm{Degradation}\;\mathrm{Ratio}\;\left(\mathrm{DR}\right)\left(\%\right)=\frac{W_{d}-W_{r}}{W_{d}}\times 100\;W_{s}W_{d}W_{r}$$

### 2.5. Rheological Characterization

All the rheological measurements were carried out in a stress-controlled rheometer (Anton Paar MCR 302, Graz, Austria). A system of parallel plates of 25 mm diameter was chosen as the geometry to perform the tests. A humidity chamber and a Peltier cell were used to avoid undesired dehumidification and to control temperature, respectively. The gap was maintained constant through all the experiments at 0.5 mm. Finally, to minimize the differences in temperature and firmness, an equilibration time of 5 min was set before the start of the test.

*Steady shear measurements*. Tests were performed in rate-controlled mode. Samples were applied a linear ramp of shear rate, $\dot{\gamma }$ starting in 0.001 to 1000 Hz at 25 °C. The resulting curves were adjusted to some of the most used and reliable models, namely, Carreau, Cross, Ostwald-de Waele model and for a more detailed analysis of the dependence between shear rate and viscosity Cross model:

(3) $$\eta =\eta_{\infty }+\frac{\eta_{0}-\eta_{\infty }}{1+K_{1}{\dot{\gamma }}^{d}}$$

where *η* represents the shear viscosity (Pa·s), *η*_0_ and *η_∞_* (Pa·s) are the no shear and the infinite viscosity (Pa·s), respectively, *K*_1_ is constant that represents the characteristic relaxation time (s), and *d* is a dimensionless constant related to the mechanical nature of the measured material.

Ostwald-de Waele model:

(4) $$\eta =K\;{\dot{\gamma }}^{n-1}$$

where *η* represents the shear viscosity (Pa·s), $\dot{\gamma }$ is shear rate (s^−1^), *K* is a constant, known as the viscosity index (Pa·s^n−1^), and n is the flow index, which, depending on the value, gives important information about the type of material. For Newtonian fluids, *n* = 1; for pseudoplastic fluids, *n* < 1; and for dilatant fluids, *n* > 1.

Carreau model:

(5) $$\frac{\eta -\eta_{\infty }}{\eta_{0}-\eta_{\infty }}={\left[1+{\left(\lambda \dot{\gamma }\right)}^{2}\right]}^{\left(n-1\right)/2}$$

where *η* represents the shear viscosity (Pa·s), *η*_0_ and *η_∞_* represent the viscosity in the first Newtonian plateau ($\dot{\gamma }$ → 0), and the second Newtonian plateau ($\dot{\gamma }$ → ∞) (Pa·s), respectively; λ is the relaxation time (s) and n is the power index.

*Dynamic measurements*. Oscillatory frequency sweep measurements were performed over frequency range of ω oscillating in a range of 0.01–200 rad/s at 25 °C. A Peltier temperature controller and a humidity chamber were used to prevent solvent evaporation and to accurately regulate the temperature of the samples. Furthermore, in order to assure that all the tests were carried out in the linear viscoelastic region (LVR), dynamic amplitude sweeps were previously completed. According to ASTM D7175 and DIN 51810-2, the point where the storage modulus G’ deviates by more than a 10% from the initial plateau, indicates that the system is no longer working with a linear viscoelastic behavior. In this case, the fixed strain chose for the frequency sweep test was 0.2%, guarantying a linear viscoelastic regime.

*Creep and recovery tests*. To obtain the recover capability of the samples, creep tests were carried out. This type of tests can be divided in two main phases. In the first one, known as the load phase, the materials underwent a constant stress of 50 Pa during 173 s. After that, the shear was removed and the response of the materials was monitored for 340 s, corresponding to the recovery phase. Results were fitted to the Burger model, which is a combination of the Maxwell model, the Kevin-Voigt model, and an empirical model, and could be expressed as follows:

(6) J(t)=1G0+1G1(1−e(−tG1η1))+1η0

**Figure 1:**
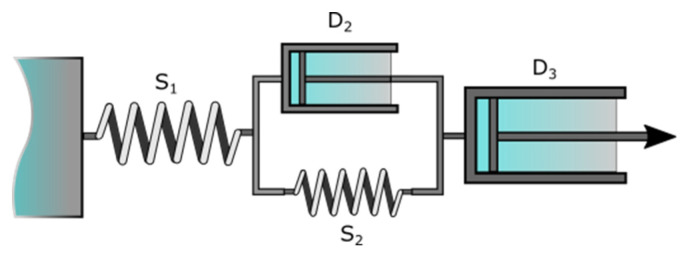
Burguer model comprising the Maxwell and Kelvin-Voigt models in series. S_1_ and D_3_ correspond to the Maxwell spring and dashpot, respectively, while S_2_ and D_2_ represent their Kelvin-Voigt counterparts.

*J*(*t*) represents the overall compliance at any time *t*. The springs correspond to Maxwell and Kelvin-Voigt (S_1_ and S_2_ in Figure 1) elastic sections with moduli *G*_0_ and *G*_1_, respectively. The dashpot of the Maxwell element (D_3_, Figure 1) is associated with the residual viscosity, *η*_0_, and the so-called internal viscosity, *η*_1_, represents the dashpot of the Kelvin-Voigt element (D_2_, Figure 1).

After the applied stress is stopped, the recovery of the systems follows a behavior that can be described with the following equation:

(7) $$J\left(t\right)=J_{\infty }+J_{KV}e^{\left(-\propto t^{\beta }\right)}$$

where *α* and *β* are parameters related to the recovery speed of the samples. *J*_∞_ is the residual deformation, Maxwell dashpot (Figure 1, D_3_) and *J_KV_* represents the delayed compliance for the Kelvin-Voigt unit. The Maxwell spring contribution is represented by J_SM_ and can be calculated as follows:

(8) $$J_{SM}=J_{MAX}-\left(J_{\infty }+J_{KV}\right)$$

where *J_MAX_* is the maximum deformation.

For a further characterization, the contribution of each of the elements can be determined as follows:

(9) $$\%\;J=\left(\frac{J_{element}}{J_{MAX}}\right)\times 100$$

Finally, the percentage of recovery of each sample after the removal of the shear can also be calculated following the equation:

(10) $$\%\;R=\left(\;\frac{J_{MAX}-J_{\infty }}{J_{MAX}}\right)\times 100$$

*Thixotropic measurements*. In order to test the thixotropic nature of the hydrogels, three interval thixotropy tests (3-ITT) were carried out. The assay involves measuring the material response to changes in the shear rate. In first place, a constant shear rate, $\dot{\gamma }$, of 0.25 Hz was applied for 10 s. After that, the shear rate, $\dot{\gamma }$, was deeply increased to 1000 Hz for 5 s. In the final step, the shear rate is set once more at 0.25 Hz and sustained for 50 s, until the end of the test. The differences in viscosity between steps give significant information about the time-dependent structure breakdown and recovery of the samples, and consequently, about their thixotropic behavior.

## 3. Results and Discussion

### 3.1. Morphological and Structural Analysis

**Figure 2:**
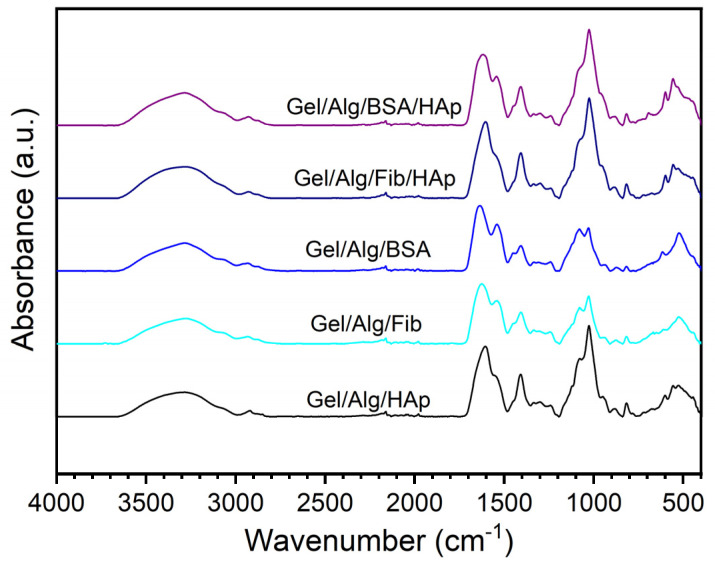
FT-IR spectra of different samples, from bottom to top: Gel/Alg/HAp, Gel/Alg/Fib, Gel/Alg/BSA, Gel/Alg/Fib/HAp, Gel/Alg/BSA/HAp.

FT-IR analysis was performed to characterize and determine the interactions and structural changes of the different components of the matrix. In order to be able to determine the effects of the components of the matrix, in the Figure 2 we show the spectra that have been obtained for samples with different composition. The characteristic peaks of alginate can be observed in the four samples: 3270 cm^−1^ (O–H stretching), 2930 cm^−1^ (C–H stretching), 1626 (C=O stretching), and 1026 (pyranosyl ring stretching). On the other hand, most of the characteristics peak of gelatin are also present in the four samples: 3069 cm^−1^ (C–H stretching), 2934 cm^−1^ (C–H stretching), 1626 (C=O stretching), and 1540 cm^−1^ (N–H stretching of secondary amide). At the same time, it is important to note that some of the peaks exhibit shifts, broadening and changes, with regard to the pure compounds, which reveals information about the structure of the samples. For example, the very broad signal on all spectra observed in the region 3000–3600 cm^−1^ correspond to a range of wavenumbers for the different O–H bonds that are uncovering the great number of hydrogen bonds present in the samples, confirming the formation of the hydrogel. Because of this broad signal some typical peaks, for example 3321 cm^−1^ (N–H stretching) in gelatin, cannot be clearly observed. Another characteristic peaks of gelatin at 1553 cm^−1^ have been shifted to 1540 cm^−1^ because of the effect of double bonds C=N and C=O. The hydrogel formation has been attributed to the reaction between aldehyde groups of alginate and amino groups of gelatin. The carbon–carbon bonds of the cis-diol groups in the molecular chain of the alginate can be cleaved to generate reactive aldehyde functions by periodate oxidation, which can develop chemical crosslinking with amino functions via Schiff‘s linkage. This fact is the cause of the enlargement of the 1626 cm^−1^ because of overlapping with the band at 1630 cm^−1^ of amide I of uncrosslinked gelatin [47]. On the other hand, the strong weakening of pure gelatin absorption peak in C–C stretching in between 1200 and 1350 cm^−1^ confirms the formation of hydrogel.

As can be seen in the Figure 2, the introduction of other components into the basic matrix of gelatin and alginate has a mild effect on the spectra. Despite this, you can see the footprint of the components. Regarding the hydroxyapatite, the peak placed at 603 cm^−1^ is attributed to the vibration of hydroxyl ions in the nanoparticles, the characteristics bands showing phosphate bending vibration in HAp can be observed at 557 cm^−1^, peak corresponding to the phosphate stretching is placed at 883 cm^−1^, being indicative of the carbonate ion substitution. Finally, the absence of the characteristic 1170 peak suggests the presence of ionic interactions between the negative charges of HAp and positive residues of gelatin mainly arginine and lysine. The addition of the proteins BSA and fibrinogen also result in small changes in the spectra. However, conformational changes can be inferred. The conformational changes of the protein secondary structure can be obtained from changes in main band in the Amide I region, at about 1655, 1672, and 1636 cm^−1^, which is assigned to α-helices, β-turn, and β-sheets, respectively. Changes in the tertiary structure are reflected in the Amide II region, 1400–1580 cm^−1^ [48]. On the other hand, the characteristic peaks of fibrinogen are placed at 1230, 1530, and 1630 cm^−1^ [49]. By comparing the spectra in Figure 2 with those of pure proteins and with the spectra without proteins, we can conclude that both proteins are perfectly integrated into the hydrogel matrix and that fibrinogen maintains its structure, while tertiary structure of BSA changes from the native form to a less compact one, probably because of interactions with gelatin and alginate chains.

**Figure 3:**
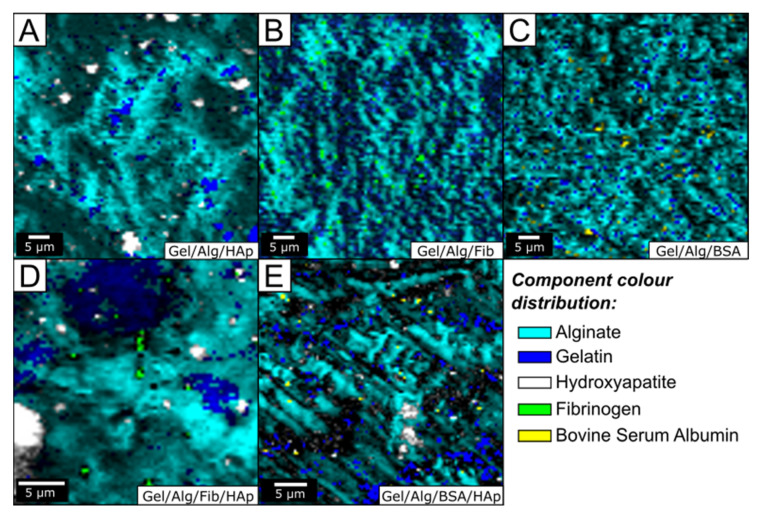
Optical images of the compound distributions of the five studied samples (wet) obtained using the integrates intensity of the Raman bands: gelatin (navy blue), alginate (dark turquoise), hydroxyapatite (white), fibrinogen (green), and BSA (yellow). (**A**) Gel/Alg/HAp, (**B**) Gel/Alg/Fib, (**C**) Gel/Alg/BSA, (**D**) Gel/Alg/Fib/HAp, (**E**) Gel/Alg/BSA/HAp.

The incorporation, in addition to the distribution, of the different components within the hydrogel matrix was further confirmed by high resolution confocal Raman microscopy. Figure 3 shows the optical micrographs where the combined maps of the individual components were identified using their specific bands: 966 cm^−1^ for hydroxyapatite, 2934 cm^−1^ for fibrinogen, and BSA and 812 cm^−1^ and 1454 cm^−1^ for alginate and gelatin respectively (we used these peaks because they shared the 2941 cm^−1^ peak, detailed spectra can be consulted in supporting material.). As shown in Figure 3, the main components of the hydrogel, gelatin, and alginate, are well distributed forming a coherent and homogeneous matrix, as it had already been predicted by FT-IR measurements. All images indicate the stability and preservation of the two initial components. Interestingly, no interference was observed, and as a result the shape of the scaffold was clearly resolved. The introduction of hydroxyapatite nanoparticles shows the profile that can be seen in the Figure 3A. The distribution of nanoparticles is not homogeneous, as can be seen, two well-differentiated sizes coexist one with and average size of 4 µm and the other with a size of 1.5 µm. The size of the hydroxyapatite nanoparticles we synthetized is of 75 nm long. So, the origin of both clusters in Figure 3A is the self-assembly of the nanoparticles. On the other hand, aggregates tend to form in gelatin-rich regions due to the electrostatic interactions with the positive residues of gelatin, as had already confirmed FT-IR analysis. Figure 3B,C show the patterns of the samples after the incorporation of fibrinogen and BSA, respectively. The images show a homogeneous distribution of both proteins throughout the scaffold. This is an important topic because to fulfill biological functions, proteins incorporated into scaffolds should maintain native conformation. Previous studies have demonstrated that fibrinogen aggregations in gelatin hydrogels are dependent on protein concentration. At high fibrinogen concentrations, the protein is forming the pore walls of the scaffolds in an aggregated state and denatured conformation, which results in a rough surface. On the contrary, at low concentrations the pore wall surface of the hydrogels appeared to be smooth and homogeneous because the reaction feed aggregation of fibrinogen was negligible [50]. Because of the low concentration of fibrinogen used in this study our results confirm that the protein is not in an aggregation state. With regard to the BSA, the pattern is similar to that of fibrinogen, previous studies found that because of the hydrophilic character and the flexibility of the molecule, it interacts positively with the scaffold favoring its incorporation into it [51]. It is important to note that the incorporation of both proteins modifies the distribution of alginate, making it much more homogeneous, suggesting that further optimization of scaffold has taken place. It is obvious that this reorganization has to do with interactions with the proteins. Interestingly, the similar morphology we observed in the hydrogel with both proteins disappears when we incorporate hydroxyapatite nanoparticles into the systems. As can be observed in Figure 3D, the presence of hydroxyapatite the fibrinogen hydrogel results in a distinct pattern with islands of higher alginate intensity, while fibrinogen and hydroxyapatite maintain the morphology they had in the original samples (Figure 3A,B). The pattern of the sample with BSA (Figure 3E) is completely different, in this case the alginate is distributed much more homogeneously, just as does the hydroxyapatite. In this case, the integration of the BSA into the scaffold, gives it the flexibility and adaptability necessary for the incorporation of hydroxyapatite nanoparticles in a homogeneous way (the morphology analysis can be consulted in Supplementary Materials, Figure S7).

### 3.2. Swelling and Degradation Behavior

In order to study the degradation of the hydrogels over time, samples were synthesized, weighed, and placed in PBS at room temperature. After the immersion, they were dried with blotting paper and weighed periodically to study their mass evolution. 

**Figure 4:**
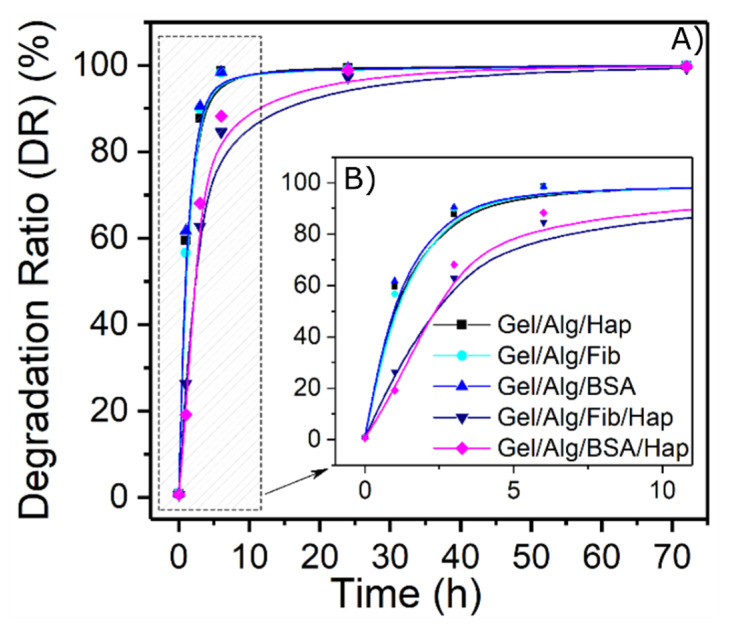
(**A**) Degradation over time of the hydrogels. (**B**) Close-up of the first 10 h of degradation.

Results from Figure 4 show that the degradation of the films occurs in a very short period of time, having a degradation over 80% in the first 5 h and over 90% within a day, for all the five samples. This mentioned degradation is a consequence of the dilution of the protein in the media, and as the protein concentration in these gels is relatively low, their degradation occurs fast. One way to delay the release is to promote the interaction of the protein ligands to form supramolecular aggregates. As can be seen in graphs, samples containing both HAp nanoparticles and proteins had an effective impact in slowing down the process. This synergistic effect is due to the formation of new bonds between the nanoparticles and the proteins which results in a decrease of the mesh size of the crosslinked network. Furthermore, as the length of the pores become smaller, the protein release is also delayed because of lower rates of diffusion.

**Figure 5:**
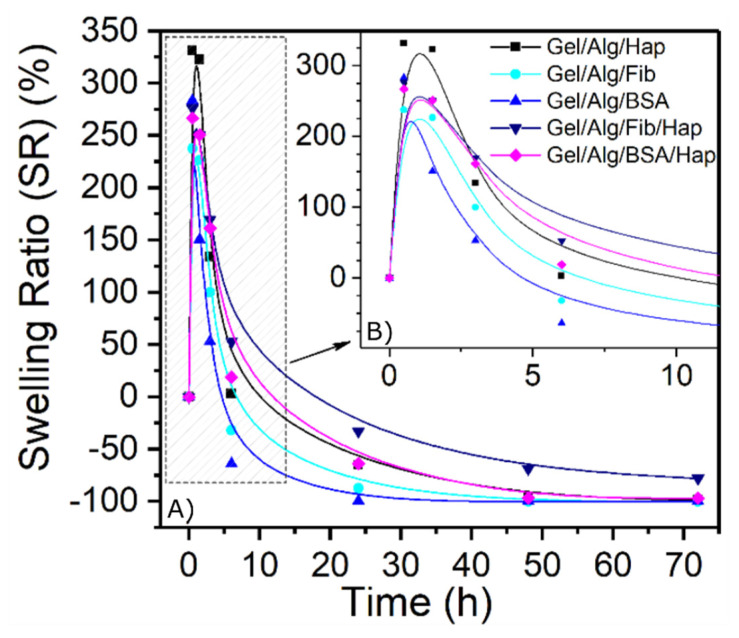
(**A**) Swelling behavior over time. (**B**) Close-up of the first 10 h of swelling.

Simultaneously, the ability to swell is also an important property of hydrogels when placed within a thermodynamically compatible solvent. To evaluate this feature, dried oven samples were weighed and then put in contact to a 37 °C PBS solution. For both cases, results demonstrated that highly cross-linked scaffolds show a facilitated entrapment of water in their framework (Figure 5), because of a narrow pore distribution which captures and holds water through capillary action. One more time, the swelling behavior of hydrogels with the incorporation of hydroxyapatite and protein are superior through time. It is well-known that nanoparticles contribute to the increase of water uptake because of its water adsorbing properties. However, in function of the results, it is proven that this effect is enhanced by the presence of proteins. This fact always may play an essential role in the steady mass loss over time found in the nanoceramic-loaded samples, turning them into better potential materials to use in guided bone regeneration.

### 3.3. Viscoelastic Properties

**Figure 6:**
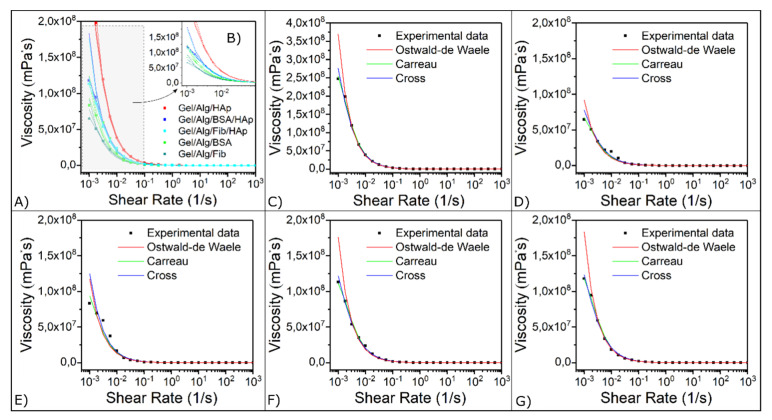
Fitting results. (**A**) Experimental and obtained data for the five studied samples. (**B**) Close up. (**C**) Gel/Alg/HAp. (**D**) Gel/Alg/Fib. (**E**) Gel/Alg/BSA. (**F**) Gel/Alg/Fib/HAp. (**G**) Gel/Alg/BSA/HAp.

Flow curve tests were performed for the five samples: giving the relationship between the sear rates, the shear stress, and the viscosity of the samples. Analyzing the graph of the viscosity against the shear stress at 25 °C (Figure 6), it can be concluded, that the different concentration of HAp nanoparticles has a noticeable impact in the viscosity values. The graphs show a rapid viscosity descent which finally stabilize into a plateau area. Moreover, in the five systems the results demonstrate that the shear thinning takes place at relatively low shear rates.

The fall of the viscosity values, as the applied stress is becoming greater, is mainly caused by the rupture of active alginate joints within the sample structure. These active joints are formed by fragmented alginate junctions, which can re-associate among them to create larger and stronger active junctions. As the concentration in nanoparticles arises, the properties and behavior of these junctions is notably modified. The presence of hydroxyapatite nanoparticles enhances the possibilities, causing the formation of new additional active joints, giving the network a higher strength and therefore higher values of viscosity as can be observed in plots Figure 6C,F,G. The effect of BSA on the matrix produces a slight improvement in strength compared to that produced by fibrinogen. This effect is preserved when hydroxyapatite nanoparticles are added. This effect is due to the greater flexibility of BSA, which guarantees the optimization of interactions, both with the original matrix and with nanoparticles.

The models described in the materials and methods section were fitted to the experimental data by means of multiple non-linear regressions. The obtained parameters are listed in Table 1. The correlation coefficient (*r*^2^) of the estimated viscosities show that the Carreau model best fits the experimental data for all samples, followed by the Cross model. On the other hand, Ostwald de Waele model also fits the experimental data but are not as good as the other two models. In the Carreau’s model, the power law index, n, characterizes the fluid behavior and values of 0 < *n* < 1, correspond to a shear thinning behavior. As could be expected all the samples exhibit shear thinning behavior. It is clear that the addition of hydroxyapatite clearly increases this index, as well as BSA containing samples results in higher values than those with fibrinogen. This increase in the index reflects the transit to a more complex internal structure (for *n* > 1 the behavior becomes shear thickening).

**Figure 7:**
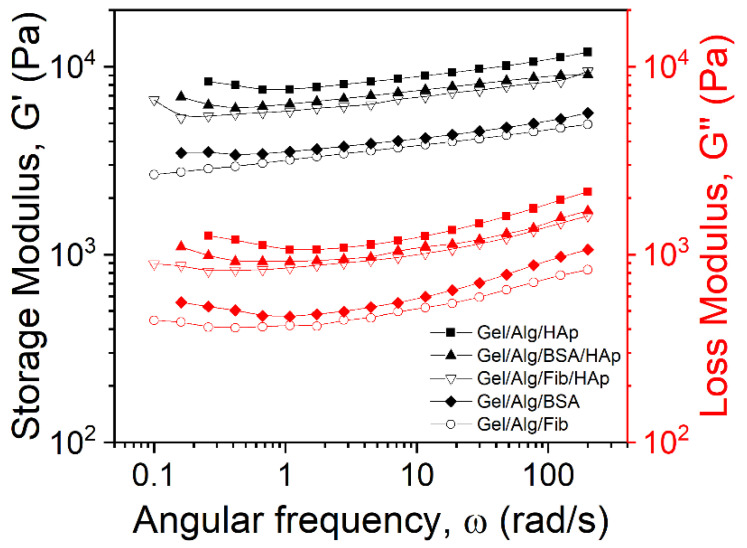
Oscillatory frequency sweep.

Analyzing the results of the frequency sweep measurements, a relationship between the viscoelastic properties of the films and the frequency can be found. At the working temperature, 25 °C the films present a gel-like behavior as the storage modulus is greater than the loss modulus [52]. This condition is the same for all the samples throughout the frequency domain studied. It can also be concluded that loss and storage moduli are dependent of the frequency, fact that has already been investigated in previous works [53]. Plots of Figure 7 are analogous to the results achieved measuring soft glassy materials with a gel structure [54]. Again, as expected, the modulus of each of the three samples presents firm nanoparticle concentration dependence. Both the loss and the storage modulus are higher in the samples were the HAp has more presence. This evidence was previously reported in different studies [5,55]. Furthermore, as it has been previously demonstrated, [56,57] the Ca atoms present on the HAp interact with the oxygen sites of alginate. The CaOH active site of HAp surface form bonds with the COO^–^ group of alginates. On the other hand, the HPO_4_^−2^ groups do not interact with the biopolymer, in particular the phosphate-hydroxyl active group [58]. Still, by gathering information, it can be assured that the alginate is successfully grafted on the hydroxyapatite surface, making the structure of the films ordered at the nanoscale range. This reinforces the hypothesis that the rheological behavior of the material corresponds to a soft glass material, as it has been previously inferred from the dependence of the moduli with the frequency. The particle bonding to the polymer network has an immediate effect on the toughness, but it is not the unique factor, as it has been proved the content of nanorods is crucial as well to the microstructures and the strength of the films [59]. One more time both proteins follow the same pattern we found previously.

**Figure 8:**
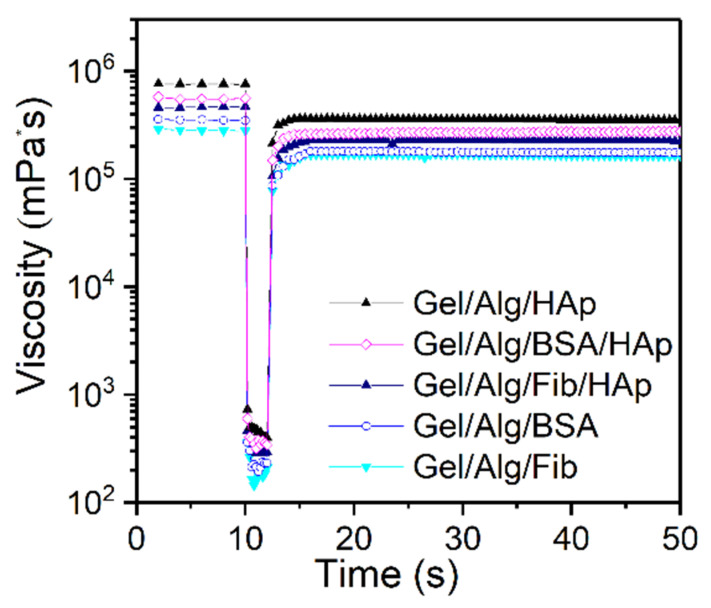
Three interval thixotropy (3-ITT) test.

Thixotropy is a time-dependent, non-Newtonian characteristic of some fluids, where the apparent viscosity decreases when a constant shearing is applied; and it rises or falls when the shear rate is changed in step. In the same way, when the shear rate is changed circularly, it causes the appearance of hysteresis loops, which is associated with the energy loss [60]. The hysteresis area is a useful tool for the estimation of the degree of thixotropy [61]. Figure 8 shows the results for the 3-ITT tests at 25 °C. The behavior is analogous for the five different samples: in the first interval, viscosity values remain constant and in the third step the material shows a reasonable thixotropic recovery, around 80%. It is commonly believed that the internal structure changes with the formation of aggregates with different crosslinking degree. When the shear rate is high enough, the network is destroyed, making the entanglements turn into monomers [62], on the other hand, when the applied shear is stopped, the structure progressively rebuilds. That means, in this case, that the rate of disentanglement and the re-entanglement is slightly different, and consequently the subtle thixotropic behavior comes out.

**Figure 9:**
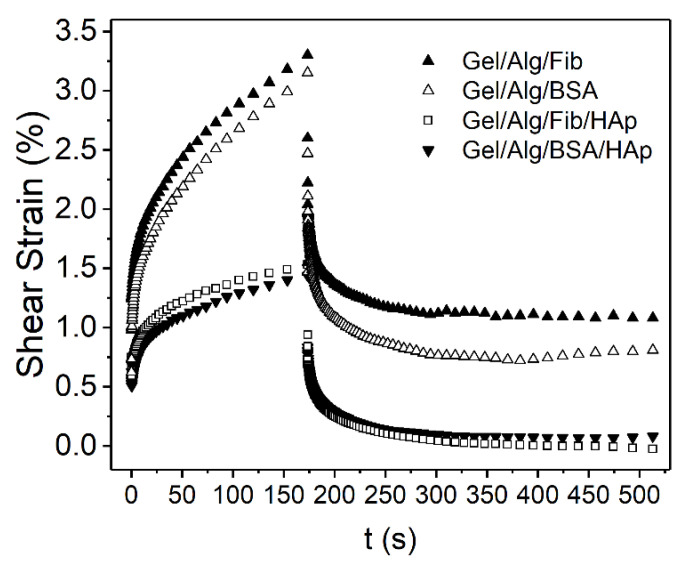
Creep-recovery curves of the four studied samples.

**Figure 10:**
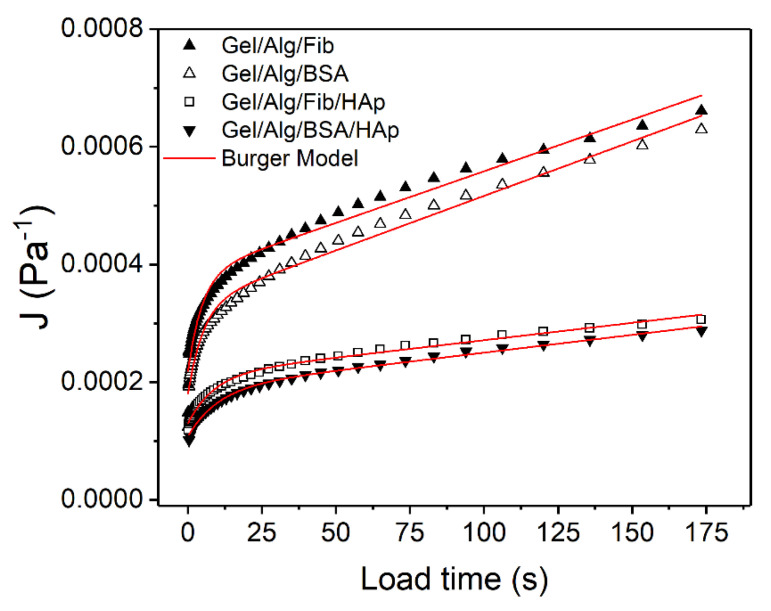
Fitted curves for the creep phase.

**Figure 11:**
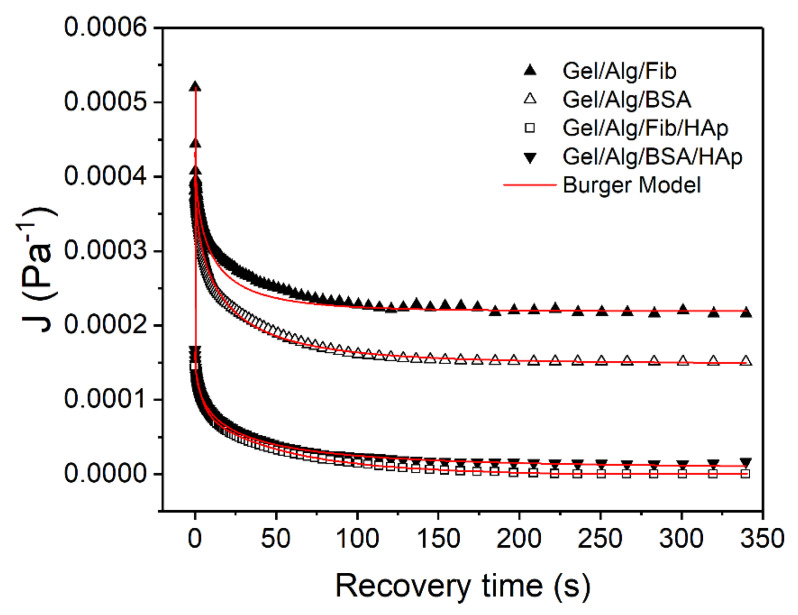
Fitted curves for the recovery phase.

Results obtained from the creep tests are shown in Figure 9. Usually, creep curves follow a typical behavior that can be divided into sections. In the load phase, immediately after the start of the test, the deformation is purely elastic, giving a jump-like response which corresponds to the spring S_1_ (see Figure 1) and represents the instantaneous compliance. After that, a delayed viscoelastic response can be observed, corresponding to the dashpot D_2_ and the spring S_2_ together, and represents the viscoelastic compliance. The values of compliance J=γ/σ, as a function of time are depicted in Figure 10 and Figure 11. The time interval for all the tests is from 0 to 173 s. The corresponding recovery, which is analyzed below, is represented for the interval 173 ≤ *t* ≤ 513 s. For the creep tests, sample Gel/Alg/HAp was not included, as the major objective of this analysis was to compare the different behavior of the gels in function of the protein added (Fib or BSA) and, at the same time, to study the influence of the nanoparticles in their response.

Burger model Equation (6) was used to fit J=f(t) in the interval 0 ≤ *t* ≤ 173 s obtaining values of *r*^2^ ≥ 0.98 in all cases. Calculated *G*_0_, *G*_1_, *η*_0_, and *η*_1_ parameters with each respective error and the correlation coefficients are shown in Table 2.

As can be seen in Table 2, *G*_0_ is lower for the gels with no presence of hydroxyapatite. Moreover, values of *η*_0_, *G*_1_, and *η*_1_ are from two to three times lower to that found for the HAp-loaded samples. This explains why the deformation seen in Figure 9 is greater for samples Alg/Gel/BSA and Alg/Gel/Fib. This fact and is in well accordance with the previously demonstrated hypothesis that the inclusion of nanorods reinforces the structure of the gels, contributing to strengthen them. Apart from that, differences found for the samples with BSA or Fib continue to corroborate previous results, suggesting that the addition of BSA exhibits a better rheological behavior.

The increase in the elastic moduli (*G*_0_ and *G*_1_) and the viscosity represented by the Maxwell dashpot can be attributed to the alignment of the nanorods to the direction of the movement on applying constant shear stress to the systems. Such movement, and the consequential redistribution of the gel network, requires and additional work, which causes an increment in the opposition to deformation. This higher resistance capacity can help to produce stability, which is an essential factor to the field of soft tissue engineering applications [63].

When the stress is stopped, σ=0, the system is in a state of maximum deformation, which is represented with *J_MAX_*. After that, the compliance values are recorded periodically in order to see the recover ability of the gels. Figure 11 reports how materials react in the interval from 173 s until the end of the experiment. To better understand and make a proper analysis, data were fitted to the Equation (7) obtaining *J_∞_* and *J_KV_*, the compliance values of the Maxwell dashpot and of the Kelvin-Voight elements, respectively. One more time, results of the proved to be sufficiently precise as *r*^2^ is close to 1. Those values, alongside *J_∞_* and *J_KV_* are presented in Table 3.

In the recovery phase, tests always follow a pattern with three different responses. The first one is represented with *J_SM_* and corresponds to the spring of the Maxwell element (S_1_). This is a practically instantaneous response which is followed by a decreasing exponential curve corresponding to the Kevin-Voight element, *J_KV_*. This response is slower and tends toward an asymptote when time tends to infinite. Finally, the permanent residual deformation corresponding to the Maxwell dashpot (D_3_), is irreversible and values are represented with *J_∞_*.

As explained, *J_MAX_* values correspond to the compliance obtained at *t* = 173 s, therefore, and using Equation (8), the compliance values corresponding to the Maxwell spring, *J_SM_* can be calculated (Table 4):

Once these parameters are known, the contribution of the Kelvin-Voight elements and the Maxwell elements to the total deformation of the systems, as well as the percentage of recovery of the gels, can be calculated through Equations (9) and (10). Results are shown in Table 5:

As can be seen, in gels with presence of HAp, the contribution of the Maxwell spring to the total deformation is heavier. Same occurs with the *J_kv_* values, meaning that the total recoverable compliance increased for these samples, which may be attributed to the increasing compactness of the structures due to the crosslinking bonds between HAp and alginate. On the contrary, the greatest contribution to the Maxwell dashpot, is found on the samples Alg/Gel/BSA and Alg/Gel/Fib, coincident with the previously described fact that those are the samples with a more liquid-like behavior.

Finally, the aforementioned increased strength of the loaded gels is again demonstrated through the values of the recovery percentage in which they reach a notably high ratio of about 97%, clear indicator that their structure, even though is more complex, is much more homogeneous and with a heavier complete response.

## 4. Conclusions

In this study we have developed the following mechanically robust hydrogel scaffolds: Gelatin/Alginate/HAp, Gelatin/Alginate/Fibrinogen, Gelatin/Alginate/BSA, Gelatin/Alginate /Fibrinogen/HAp, and Gelatin/Alginate/BSA/HAp; all of them exhibiting a highly interconnected mesh structure as was confirmed by FT-IR. Confocal Raman microscopy revealed that both proteins are incorporated into the scaffolds while maintaining its native conformation. In addition, the presence of proteins contributes to a better distribution of alginate in the matrix, suggesting that further optimization of scaffold has taken place. The presence of hydroxyapatite causes different morphological patterns to occur while fibrinogen gels maintain initial distributions, the presence of BSA homogenizes much better all components (alginate, hydroxyapatite, and BSA). This fact is attributed to the greater flexibility of the BSA that allows more degrees of freedom to the scaffold to minimize the energy of the hydrogel formation. Besides, Gel/Alg/BSA/HAp hydrogel exhibited better degradation and swelling properties through time because of the better distribution of HAp which contribute to the increase of water uptake because of its water-adsorbing properties. Rheological analysis reveals that the behavior of the materials corresponds to a soft glass material. Creep analysis demonstrated that the inclusion of nanorods reinforces the structure of the gels, contributing to strengthen them and corroborated that the addition of BSA exhibits a better rheological behavior. Finally, we have demonstrated that the morphological and mechanical properties of the hydrogels can be easily tuned by including several components, therefore providing better performances to soft tissue engineering applications.

## Figures and Tables

**Table 1:** Flow curve modeling.

Samples						
	Parameters	Gel/Alg/Fib	Gel/Alg/BSA	Gel/Alg/Fib/HAp	Gel/Alg/BSA/HAp	Gel/Alg/HAp
Ostwald-de Waele	*K*	148.31	170.09	207.17	212.11	351.63
	*n*	0.0692	0.0542	0.0240	0.0213	0.0068
	*r* ^2^	0.88	0.87	0.80	0.81	0.85
Carreau	*η*_0_	8.44 × 10^7^	1.29 × 10^8^	1.32 × 10^8^	1.41 × 10^8^	3.25 × 10^8^
	*η_∞_*	57.688	83.806	91.906	95.432	98.690
	*λ*	784.61	968.68	641.72	666.37	787.3
	*n*	0.4753	0.4830	0.4960	0.4969	0.5096
	*r* ^2^	0.98	0.95	0.99	0.99	0.99
Cross	*η*_0_	2.00 × 10^8^	2.14 × 10^8^	2.78 × 10^8^	2.68 × 10^8^	7.87 × 10^8^
	*η_∞_*	69.151	85.956	96.405	92.515	99.621
	*K*_1_	1606.6	6396.6	1294.2	1176.9	1837.0
	*d*	0.9631	0.9727	0.9951	0.9978	1.0188
	*r* ^2^	0.96	0.94	0.99	0.99	0.98

**Table 2:** Values of the Burger model parameters. Results from the fits by Equation (6).

	*G*_0_ × 10^−3^ (Pa)	*η*_0_ × 10^−4^ (Pa·s)	*G*_1_ × 10^−3^ (Pa)	*η*_1_ × 10^−4^ (Pa·s)	*r* ^2^
**Gel/Alg/BSA**	5.62 ± 0.21	53.95 ± 2.22	6.52 ± 0.34	3.32 ± 0.49	0.986
**Gel/Alg/Fib**	4.64 ± 0.19	56.99 ± 2.81	5.97 ± 0.35	2.86 ± 0.47	0.980
**Gel/Alg/BSA/HAp**	9.29 ± 0.11	165.94 ± 5.32	12.21 ± 0.29	12.45 ± 6.95	0.997
**Gel/Alg/Fib/HAp**	7.75 ± 0.10	169.31 ± 8.17	12.01 ±0.40	10.51 ± 8.27	0.992

**Table 3:** Compliance of the Maxwell dashpot, Kevin-Voigt element and parameters obtained from the fitting by Equation (7).

	*J_∞_* × 10^5^ (Pa^−1^)	*J_KV_* × 10^5^ (Pa^−1^)	*α* (s^−1^)	*β*	*r* ^2^
**Gel/Alg/BSA**	14.88 ± 0.13	25.08± 0.34	0.21 ± 0.01	0.56 ± 0.01	0.997
**Gel/Alg/Fib**	21.92 ± 0.37	22.69 ± 1.35	0.31 ± 0.05	0.54 ± 0.05	0.953
**Gel/Alg/BSA/HAp**	0.79 ± 0.08	14.63 ± 0.16	0.25 ± 0.01	0.48 ± 0.01	0.999
**Gel/Alg/Fib/HAp**	0.87 ± 0.03	13.88 ± 0.49	0.16 ± 0.02	0.59 ± 0.03	0.989

**Table 4:** Maximum compliance and compliance of the Maxwell spring.

	*J_MAX_* × 10^5^ (Pa^−1^)	*J_SM_* × 10^5^ (Pa^−1^)
Gel/Alg/BSA	56.965 ± 0.01	17.006 ± 0.51
Gel/Alg/Fib	66.094 ± 0.01	21.490 ± 1.71
Gel/Alg/BSA/HAp	28.726 ± 0.01	13.309 ± 0.23
Gel/Alg/Fib/HAp	30.624 ± 0.01	15.875 ± 0.61

**Table 5:** Percentage contribution of the Burger elements to the total recovery of the systems.

	*J_SM_* (%)	*J_KV_* (%)	*J_∞_* (%)	*R_G_* (%)
Gel/Alg/BSA	29.85	44.03	26.12	73.88
Gel/Alg/Fib	32.51	34.32	33.16	66.84
Gel/Alg/BSA/HAp	46.33	50.92	2.75	97.25
Gel/Alg/Fib/HAp	51.84	45.33	2.83	97.16

## Supplementary Materials

The following are available online at https://www.mdpi.com/2079-4991/10/7/1302/s1, Figure S1. Raman spectra of the different compounds of the hydrogels; Figure S2. Gel/Alg/HAp hydrogel. Up, Compound distribution. Middle, single compounds. Bottom, surface map. Dark turquoise represents alginate distribution, navy blue gelatin and white Hydroxyapatite, respectively; Figure S3. Gel/Alg/Fib hydrogel. Up, Compound distribution. Middle, single compounds. Bottom, surface map. Dark turquoise represents alginate distribution, navy blue gelatin and green fibrinogen, respectively; Figure S4. Gel/Alg/BSA hydrogel. Up, Compound distribution. Middle, single compounds. Bottom, surface map. Dark turquoise represents alginate distribution, navy blue gelatin and yellow BSA, respectively; Figure S5. Gel/Alg/Fib/HAp hydrogel. Up, Compound distribution. Middle, single compounds. Bottom, surface map. Dark turquoise represents alginate distribution, navy blue gelatin, green fibrinogen and white Hydroxyapatite, respectively; Figure S6. Gel/Alg/BSA/HAp hydrogel. Up, Compound distribution. Middle, single compounds. Bottom, surface map. Dark turquoise represents alginate distribution, navy blue gelatin, yellow BSA and white Hydroxyapatite, respectively; Figure S7. 3D surfaces of the studied samples. The root mean square roughness (Rq), roughness arithmetical average deviation (Ra), Skewness (Rsw) and Kurtosis (Rku) coefficients of these samples can be consulted in the Table below; Table S1. Root mean square roughness (Rq), roughness arithmetical average deviation (Ra), Skewness (Rsw) and Kurtosis (Rku) coefficients of the studied samples.
