# Supplementary material for: Soft Actuated Hybrid Hydrogel with Bioinspired Complexity to Control Mechanical Flexure Behavior for Tissue Engineering
Source: Nanomaterials (Basel). 2020 Jul 3;10(7):1302. doi: 10.3390/nano10071302 (PMC7407768; doi:10.3390/nano10071302)
Supplement: Supplementary file 1 [file nanomaterials-10-01302-s001.pdf]

Supplementary Materials

Soft Actuated Hybrid Hydrogel with Bioinspired Complexity to Control Mechanical Flexure Behavior for Tissue Engineering

Ramón Rial [1], Zhen Liu [2] and Juan M. Ruso [1,*]

[1] Soft Matter and Molecular Biophysics Group, Department of Applied Physics, University of Santiago de Compostela, 15782 Santiago de Compostela, Spain; ramon.rial@usc.es

[2] Department of Physics and Engineering, Frostburg State University, Frostburg, MD 21532, USA; zliu@frostburg.edu

* Correspondence: juanm.ruso@usc.es

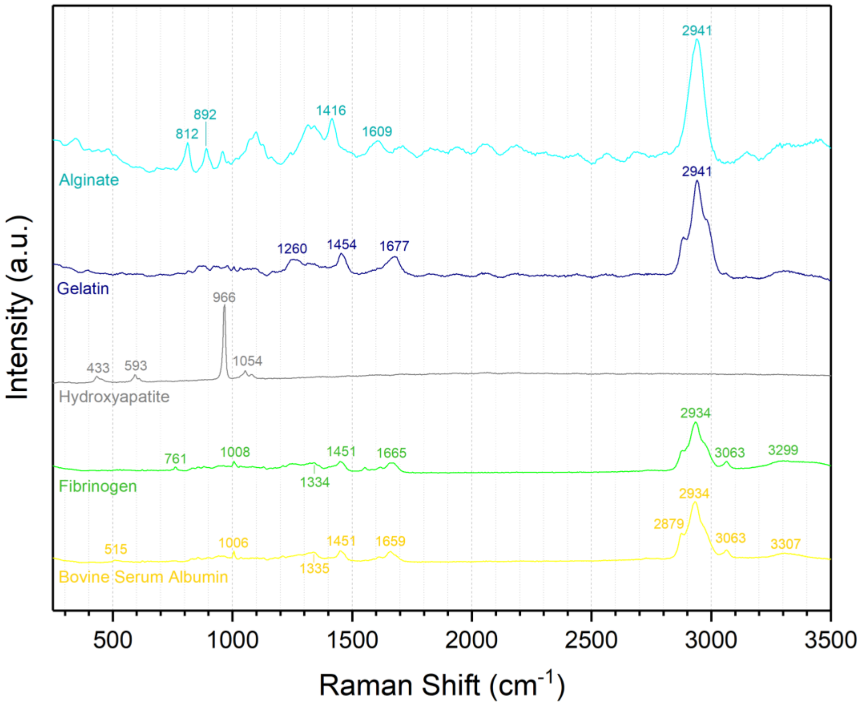

Figure S1. Raman spectra of the different compounds of the hydrogels.

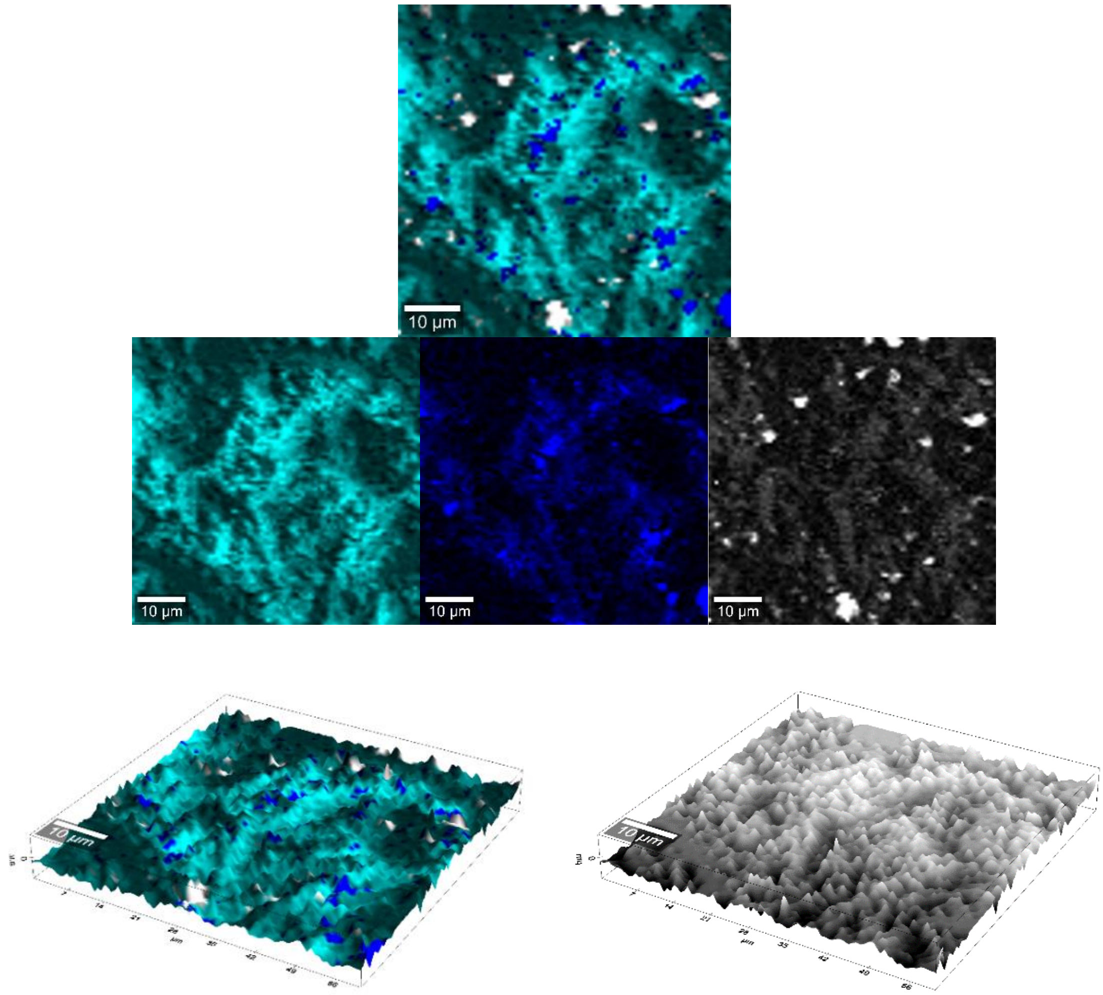

Figure S2. Gel/Alg/HAp hydrogel. Up, Compound distribution. Middle, single compounds. Bottom, surface map. Dark turquoise represents alginate distribution, navy blue gelatin and white Hydroxyapatite, respectively.

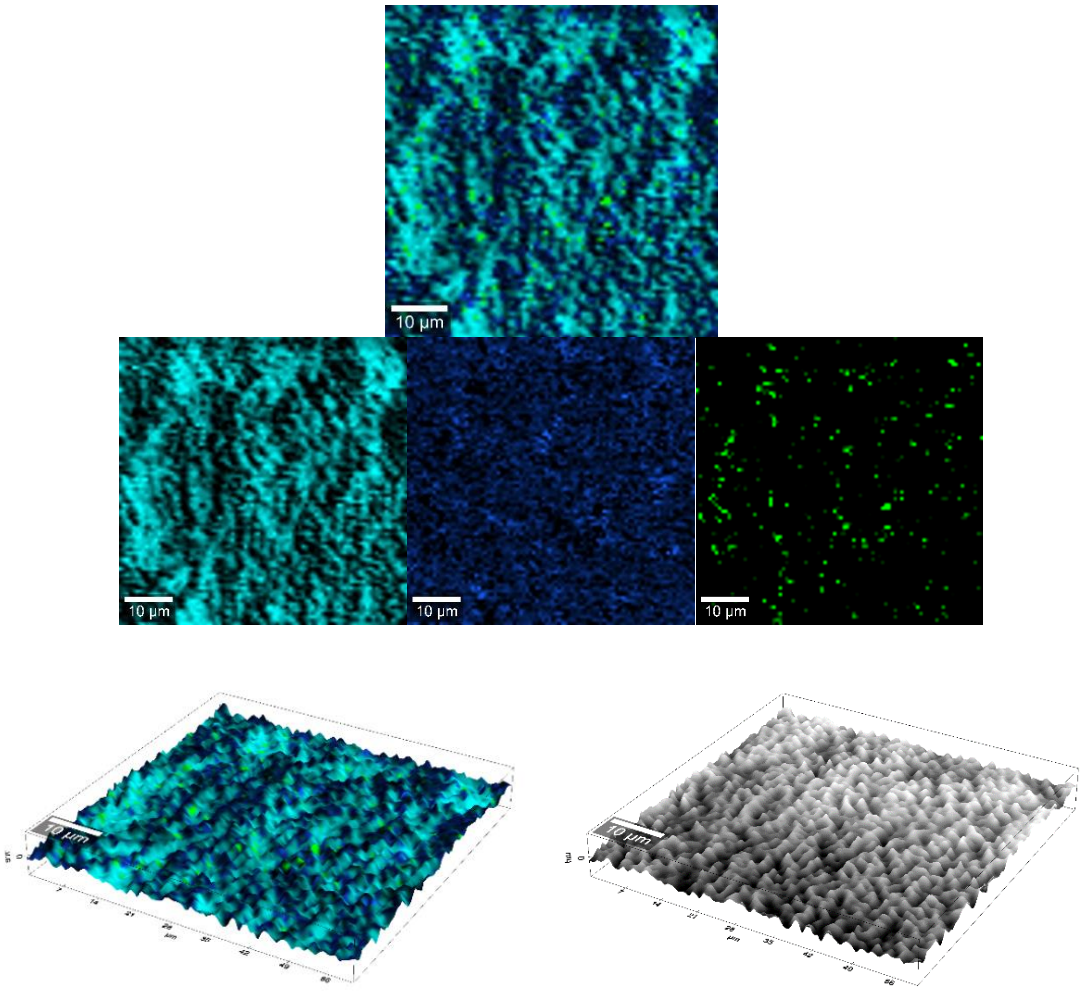

Figure S3. Gel/Alg/Fib hydrogel. Up, Compound distribution. Middle, single compounds. Bottom, surface map. Dark turquoise represents alginate distribution, navy blue gelatin and green fibrinogen, respectively.

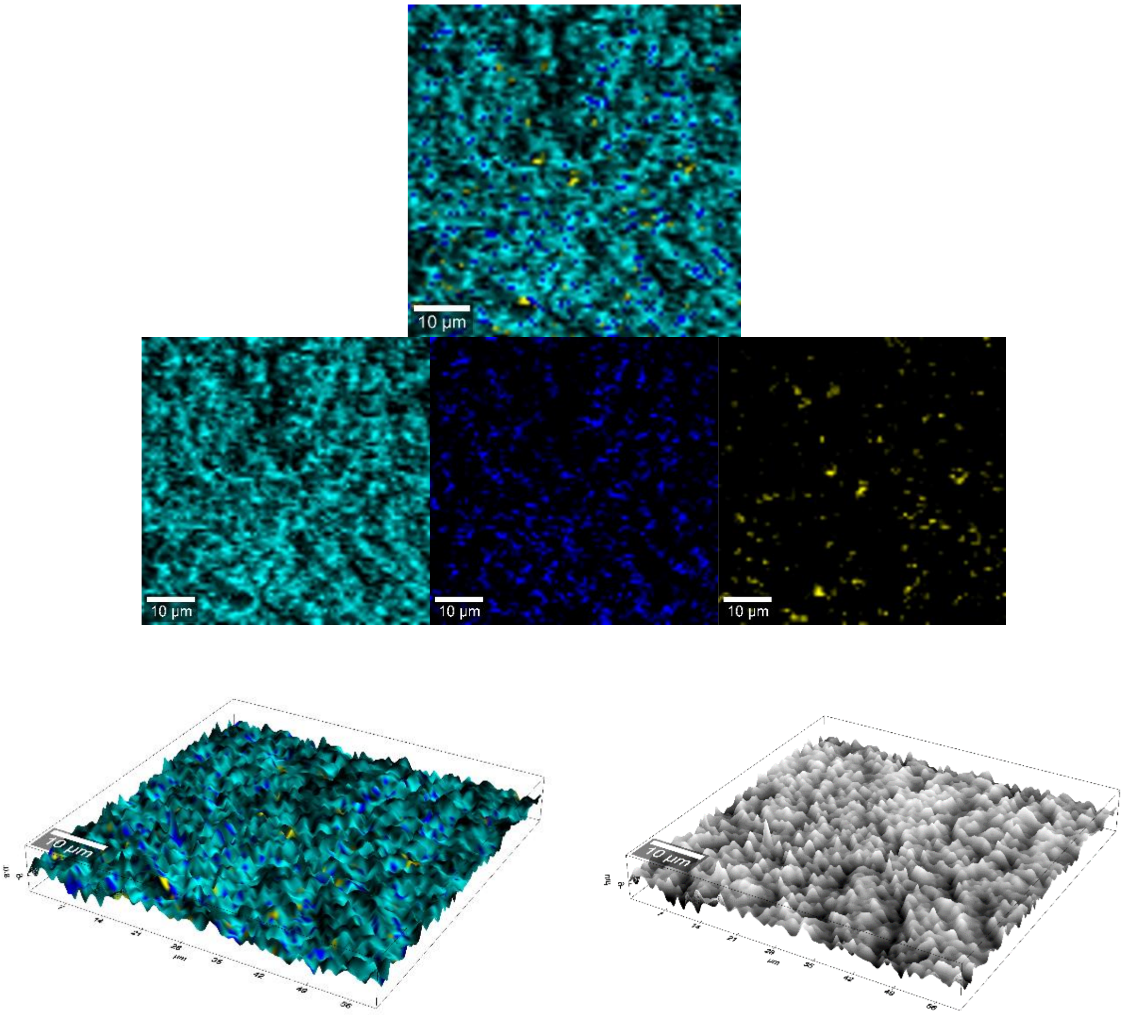

Figure S4. Gel/Alg/BSA hydrogel. Up, Compound distribution. Middle, single compounds. Bottom, surface map. Dark turquoise represents alginate distribution, navy blue gelatin and yellow BSA, respectively.

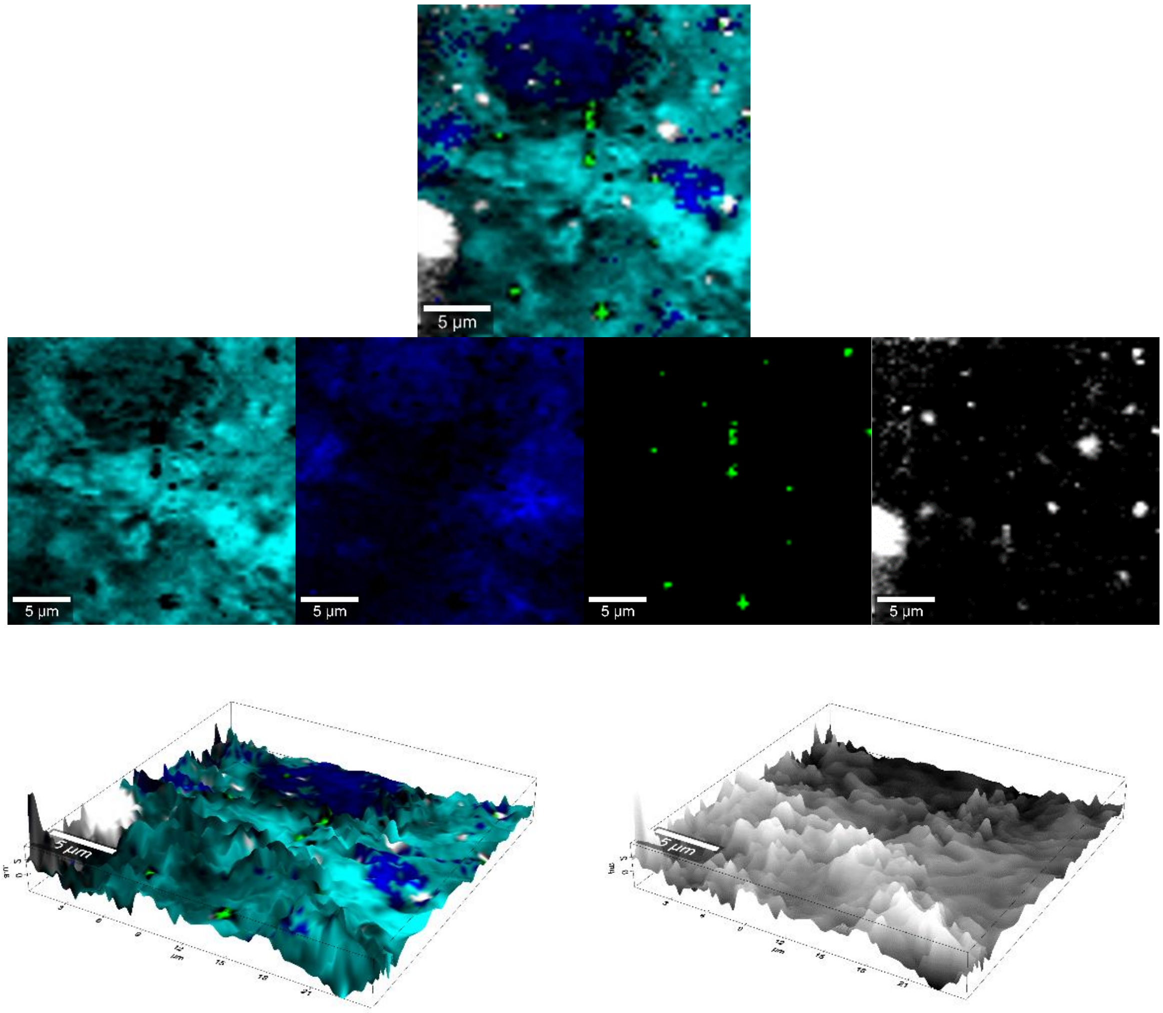

Figure S5. Gel/Alg/Fib/HAp hydrogel. Up, Compound distribution. Middle, single compounds. Bottom, surface map. Dark turquoise represents alginate distribution, navy blue gelatin, green fibrinogen and white Hydroxyapatite, respectively.

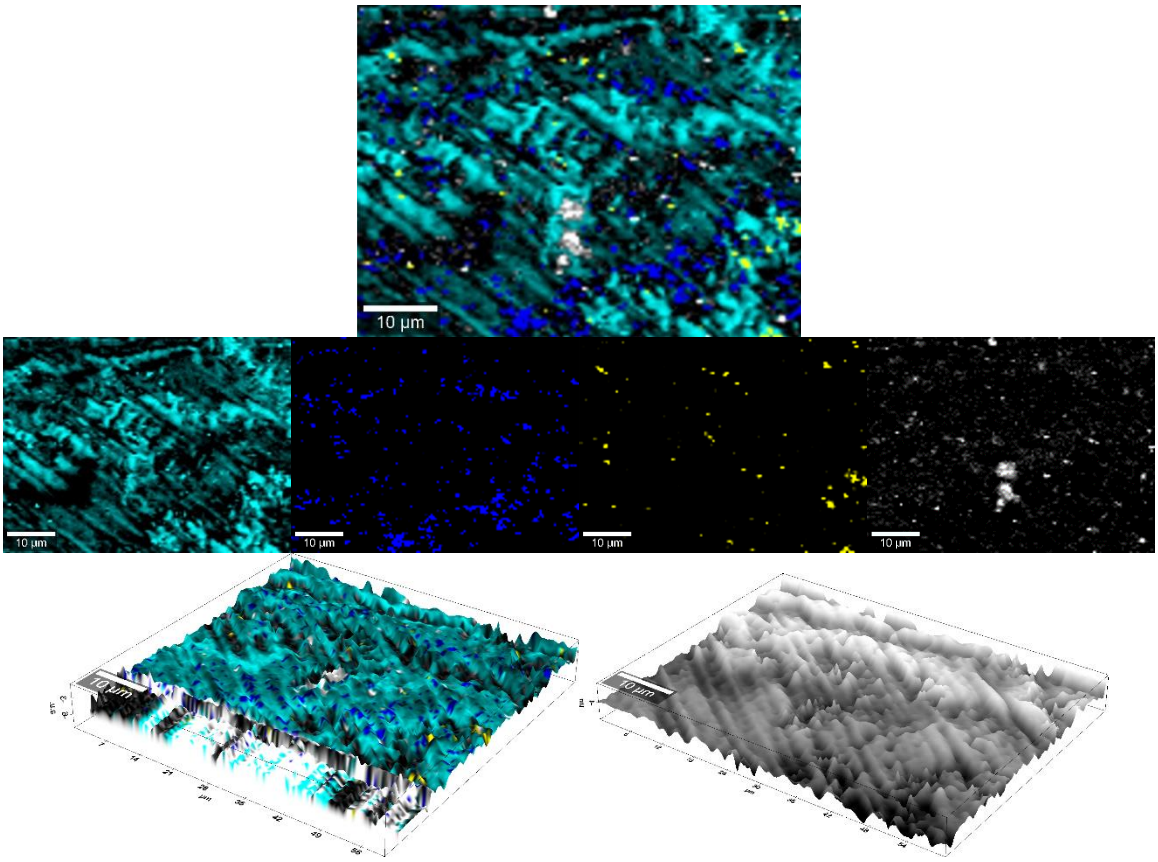

Figure S6. Gel/Alg/BSA/HAp hydrogel. Up, Compound distribution. Middle, single compounds. Bottom, surface map. Dark turquoise represents alginate distribution, navy blue gelatin, yellow BSA and white Hydroxyapatite, respectively.

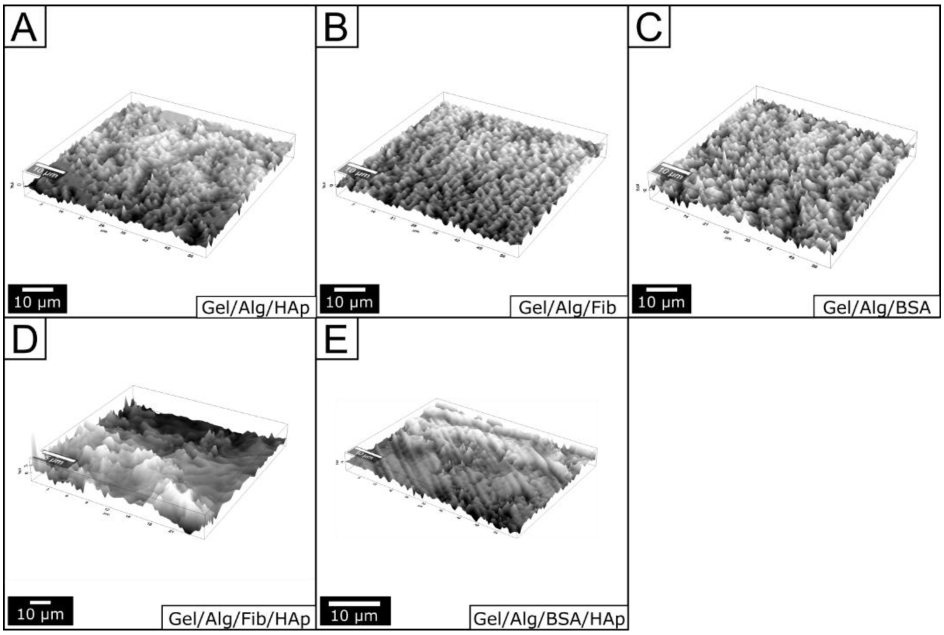

Figure S7. 3D surfaces of the studied samples. The root mean square roughness (Rq), roughness arithmetical average deviation (Ra), Skewness (Rsw) and Kurtosis (Rku) coefficients of these samples can be consulted in the Table below.

Table S1. Root mean square roughness (Rq), roughness arithmetical average deviation (Ra), Skewness (Rsw) and Kurtosis (Rku) coefficients of the studied samples

	R_q	R_a	R_{sw}	R_{ku}
Sample A	225.9	213.3	1.09	1.21
Sample B	235.5	228.1	1.06	1.13
Sample C	235.2	228.6	1.05	1.12
Sample D	235.3	227.4	1.06	1.13
Sample E	211.8	199.6	1.15	1.30

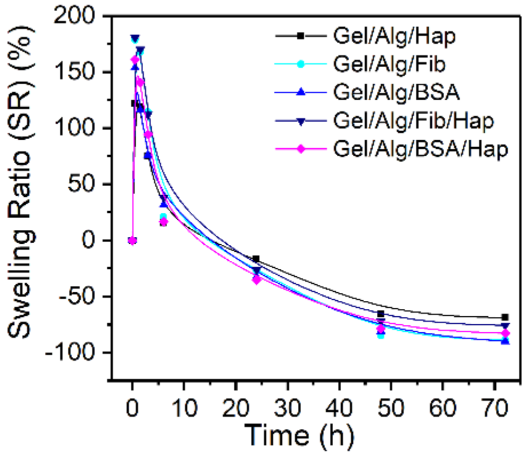

Figure S8. Swelling behaviour over time. Lyophilized samples.

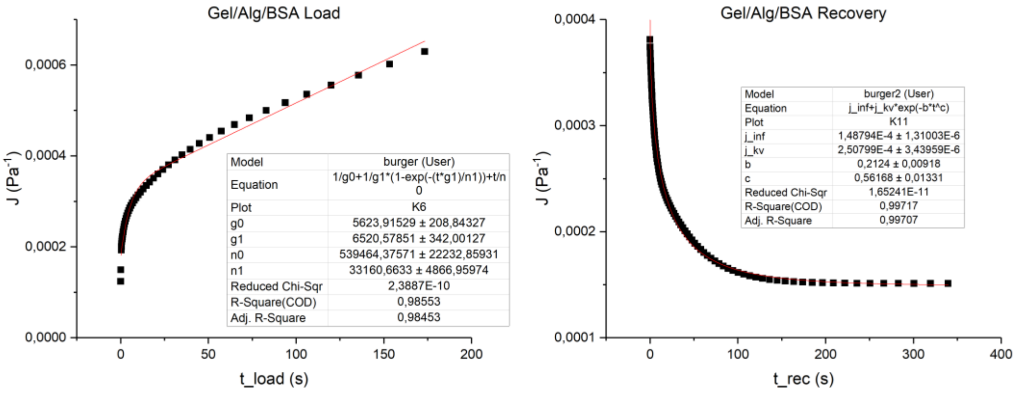

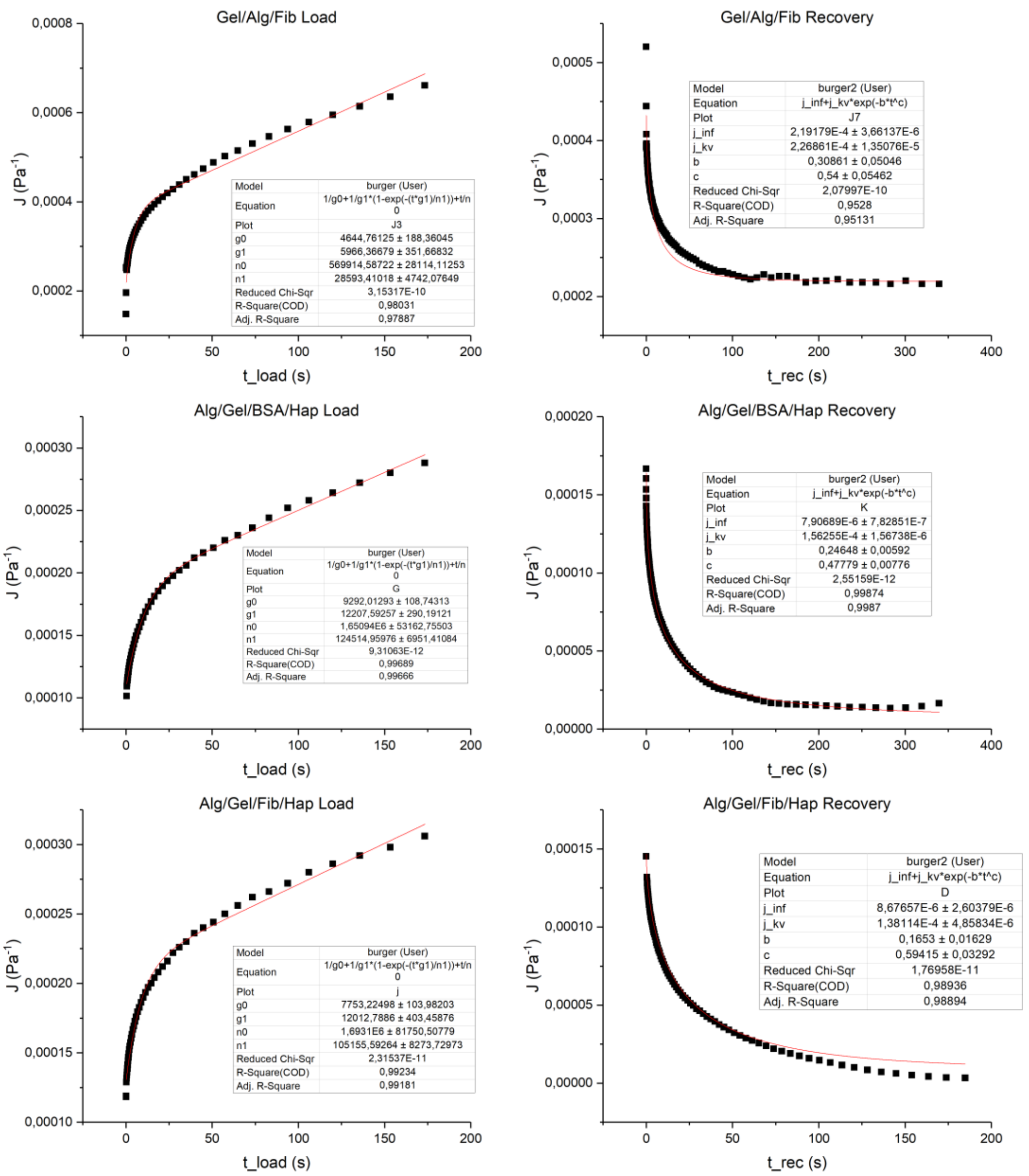

Figure S9. Fitting results for the Burger model in the creep tests. Load and recovery phases are depicted with each respective fitting equation.
